# Effectiveness of Six Sigma in Managing Medical Errors in the Electronic Drug Administration Record (EDAR) System

**DOI:** 10.7759/cureus.84864

**Published:** 2025-05-26

**Authors:** Duaa A Ashgar, Noot M Alotaibi, Saad M Al-Shahrani, Sarah Almanea, Abdulaziz S Almulhim, Alsayed Alharbi, Nourah Alhudaithi, Ameera Attia, Yasser A Alghamdi, Duaa Aljabri

**Affiliations:** 1 Continuous Quality Improvement and Patient Safety Administration Department, Armed Forces Hospital, Dhahran, SAU; 2 Pharmaceutical Department, Armed Forces Hospital, Dhahran, SAU; 3 Medical Administration Department, Armed Forces Hospital, Dhahran, SAU; 4 Health Information Management and Technology Department, Imam Abdulrahman Bin Faisal University, Dammam, SAU

**Keywords:** electronic data, medical errors (mes), patients' safety, prescriptions, six sigma

## Abstract

Introduction: Medication errors can occur at any stage of the medication process, including prescribing, preparing, dispensing, or administering the medication by nurses. Any of these medication errors will impact patient safety and healthcare quality.

Aim: This study aimed to evaluate the effectiveness of the Six Sigma methodology in reducing medication errors within the Electronic Drug Administration Record (EDAR) system at the Armed Forces Hospital in Dhahran, Saudi Arabia.

Methods: An assessment and the key elements of the Institute for Safe Medication Practices for medication use system were applied to the medication system to identify our pain points. A multidisciplinary team utilized the Six Sigma methodology following the phases of Define, Measure, Analyze, Improve, and Control framework over a 12-month period. Before intervention, data on prescribing errors were collected from the incident report system and compared retrospectively to all inpatient prescriptions from the EDAR pharmacy system. Prescribing errors analysis revealed a baseline error rate set at 2.1%. Key performance indicators were established to measure the effectiveness of implemented interventions.

Results: Implementing targeted interventions resulted in a significant reduction in inpatient prescribing errors, achieving a new error rate of 1.3% and surpassing the initial target of ≤1.9%. The observed errors decreased from 2.1% to 1.3%, and the Sigma level improvement from 3.5 to 3.7 indicates a substantial enhancement in process performance, suggesting a true impact of the intervention. The defects per million opportunities improved from 20,532 to 13,760, reflecting a shift in the Sigma level from 3.5 to 3.7. Feedback from stakeholders indicated improved communication and user satisfaction with the EDAR system.

Conclusion: The study demonstrates that Six Sigma methodology effectively reduces medication errors and enhances patient safety within healthcare settings. Continued investment in optimizing pharmacy systems and fostering a culture of safety is crucial for sustaining these improvements and advancing the quality of patient care.

## Introduction

Prescription errors represent a serious concern within the medication use process, as they pose substantial risks to patient safety and the quality of care delivered [1]. Prescription errors are mistakes that occur in the process of communicating medication information and instructions between prescribers and pharmacists, often leading to incorrect, incomplete, or unclear prescriptions [2]. The Institute of Medicine estimates that there are approximately 51.5 million prescription errors annually in the United States alone, based on three billion prescriptions written each year [3]. A systematic review of prescribing errors in England revealed that 50% of medication errors occur during hospital admissions, further underscoring the prevalence of this issue [4]. Moreover, in the United States and Europe, prescription errors are believed to be the third leading cause of mortality [5].

Globally, medication errors and unsafe drug practices are among the leading causes of preventable harm and injury in healthcare systems [6]. The consequences of these errors can range from temporary disability and prolonged hospitalization to life-threatening conditions, birth defects, or even death [7]. The World Health Organization estimates that the annual cost of medication errors is approximately USD 42 billion, representing almost 1% of global healthcare expenditures [6,8].

In Saudi Arabia, empirical data on medication errors are limited. One study conducted in a major tertiary care setting found that medication errors occurred at a rate of 1.5 per 100 prescriptions, with one-third of adverse drug reactions attributed to these errors [9]. Another study highlighted that most reported errors were near misses and preventable prescribing errors [10]. A meta-analysis by Almalki et al. confirmed that prescription errors are the most prevalent type of medication error in Saudi hospitals, accounting for 44.4% of all errors [11]. These findings underscore the need for more comprehensive studies in Saudi Arabia to better understand the scale and implications of medication errors and to develop effective strategies for preventing them. While prescription errors are a global concern, the lack of detailed empirical research in Saudi Arabia highlights the need for further studies to quantify the prevalence of medication errors and their consequences. Such research would provide essential insights to inform targeted interventions. A recent systematic review of 28 studies on medication errors in Saudi Arabia revealed that wrong doses, improper doses, and prescribing errors are the most prevalent, highlighting an urgent need for targeted interventions [10].

Accreditation bodies advocate for healthcare organizations to track and evaluate performance indicators related to prescribing errors, medication safety incidents, and adverse drug events to drive continuous improvement and elevate patient safety standards. Internationally, esteemed organizations like the Institute for Safe Medication Practices (ISMP) and the National Coordinating Council for Medication Error Reporting and Prevention (NCC-MERP) strongly advise against using medication error rates as benchmarks for performance or patient safety [2]. Instead, they emphasize a multifaceted approach that includes fostering a safety culture, enhancing reporting mechanisms, and improving processes to evaluate medication safety programs effectively [12,13]. Nationally, the Central Board of Accreditation for Healthcare Institutions (CBAHI) and the Joint Commission International (JCI) set key policies to address prescription errors within the framework of patient safety and quality care. CBAHI’s standard MM.41 mandates hospitals to implement systems for reporting and managing medication errors, highlighting the importance of proactive risk management strategies such as regular medication reviews, standardizing prescribing procedures, and staff training (MM.41 standard) [14]. Similarly, JCI’s standard MMU.7.1 focuses on protocols for reporting and addressing medication errors and near misses, emphasizing adherence to evidence-based prescribing guidelines to reduce variability, mitigate errors, and enhance patient outcomes (MMU.7.1) [15]. This standard is also part of the International Patient Safety Goals required for hospital certification by JCI.

The review identified a lack of empirical studies examining the effectiveness of electronic drug administration systems in Saudi Arabia. While interventions such as electronic prescribing and optimizing pharmacist-to-patient ratios in critical care are encouraged, more empirical research is needed to assess their real-world impact. Furthermore, the potential of advanced quality management strategies like Six Sigma in managing and reducing medical errors remains underexplored. Investigating the role of Six Sigma and similar interventions could provide valuable insights into improving error management. In this context, this study aims to investigate the impact of Six Sigma methodology in reducing medical errors in the Electronic Drug Administration Record (EDAR) system.

Background

A recent analysis conducted at the Armed Forces Hospital (study setting) in Dhahran, Saudi Arabia, revealed that prescribing errors accounted for 83% of all medication errors, making them the most dominant type of error in the medication process. Appendix 1 shows the percentage distribution of medication errors by stages of the medication process for the last three months of 2022. Appendices 2, 3 illustrate the incidence of prescribing errors as a percentage for inpatient and outpatient prescriptions over six months, from quarter 4 (2022) to quarter 1 (2023). The overall incidence of prescribing errors was 0.4% for inpatient prescriptions and 0.5% for outpatient prescriptions, as detailed in Appendix 3. Specifically, Appendix 3 provides a more detailed distribution of errors among all prescriptions, showing that inpatient prescribing errors constituted 2.1% of all inpatient prescriptions, while outpatient prescribing errors represented 0.6% of total outpatient prescriptions. These findings highlight the critical need for identifying and implementing strategies to enhance the medication use system, ultimately aiming to reduce the risk of future errors and safeguard patient safety.

By focusing on root causes and implementing proactive strategies, healthcare organizations can significantly reduce medication errors, improve patient safety, and elevate the quality of care. Various factors contributing to medication errors include healthcare workers, patients, the work environment, and issues related to computerized systems and care transitions [16]. This study aims to assess how well the Six Sigma methodology works by using the ISMP guide's preventive or corrective steps to find, stop, and eliminate medication errors at the Armed Forces Hospital, to prevent patient harm from these prescribing mistakes.

## Materials and methods

Study setting and context

The project was commenced in the Armed Forces Hospital in Dhahran Inpatient Pharmacy, Saudi Arabia, during the year 2022 to 2024.

Study design

The project team selected the Six Sigma methodology to be applied in the project, which follows the Define, Measure, Analyze, Improve, and Control dynamic approach. Six Sigma is a quality improvement (QI) methodology that aims to optimize operations while lowering defects and expenses. It was invented and is widely used in industry, and it has lately been adopted, on a small basis, in healthcare [17]. This approach demonstrated a substantial reduction in medication mistakes in the outpatient pharmacy department of a Saudi Arabian hospital [18,19]. The "Sigma" level in Six Sigma is a statistical measure of process performance that indicates how much variance occurs in a process when compared to its established criteria or specifications. It is commonly stated as Defects Per Million Opportunities (DPMO) [17].

Inclusion and exclusion criteria

The inclusion criteria were all inpatient prescriptions processed through the EDAR system within the study timeframe (from October 2022 to March 2024) as well as all the incidents of prescribing errors reported during the intervention and control phases of the study, and participants (healthcare professionals) involved in the medication prescribing, dispensing, and administration process who consented to contribute data for analysis. Hence, the exclusion criteria were outpatient prescriptions, as the focus was on inpatient error reduction, prescriptions without complete documentation in the EDAR system, emergency prescriptions where the standard prescribing process was bypassed due to critical time constraints, and Healthcare professionals or prescriptions outside the scope of the EDAR system during the study period.

Ethics

Ethical approval was obtained from the Institutional Review Board (IRB) of Armed Forces Hospitals Eastern Province, IRB number: AFHER-IRB-2025-014.

Define phase

The define phase is used to establish a solid understanding of the scope and aim. During this phase, the project's objectives and scope will be specified. One of the most important success factors in a Six Sigma project is a comprehension of what service processes are required to fulfill the objectives. During the define phase, baseline data were collected to determine the prevalence of medication errors at the hospital. The nature of the problem was discovered using QI tools (flowchart, suppliers, inputs, process, outputs, customers, SIPOC, and stakeholder analysis), which revealed that errors occurred more frequently in patient prescriptions.

**Figure 1:**
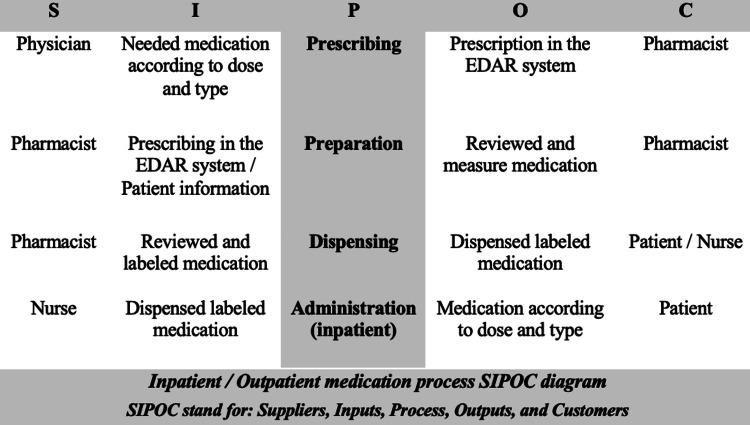
Inpatient and outpatient medication process SIPOC diagram EDAR: Electronic Drug Administration Record

To understand the nature of the problem, the flowchart of the medication process has been designed to identify errors and map each step, starting from prescription by a physician, prescription by a pharmacist, dispensing, and administration by a nurse. In the SIPOC diagram, as shown in Figure 1, the supplier and the customer have been defined to further define the process boundaries with the stakeholders. Then, stakeholder position analysis was applied to understand the needs and expectations of all the team members. For this study, a multidisciplinary team was established to ensure a comprehensive approach to addressing medication errors, leveraging expertise from various hospital departments. The Process Improvement Specialist took on the role of Team Leader, applying Six Sigma tools to guide the improvement process, while the Pharmacy Quality Coordinator/Medication Safety Officer served as the Co-Team Leader, focusing on medication safety aspects. Other key members included the Director of Internal Medicine and an Information Technology (IT) Programmer, who participated as Team Members, providing clinical and technical expertise. A second Process Improvement Specialist functioned as the Facilitator, ensuring smooth coordination and application of process improvement methodologies across the team.

Measure phase

In the Measures phase, we set the data collection plan using the key performance indicator (KPI) cards. All KPI cards include all specification elements for the seven key performance measures. We set the data collection plan using KPI cards that include all specification elements, and a data collection sheet (Microsoft Excel dashboard, Microsoft Corporation, Redmond, WA) was designed. Then, we measured the DPMO calculation. The defects were determined by dividing inpatient prescribing errors by the total inpatient prescriptions for the corresponding period. The number of opportunities will be equal to one, due to any reported prescribing error being considered as one defect. DPMO was calculated at 3.5 sigma level.

$DPMO=\frac{521}{25,375&times;1}&times;1000,000=20,532.097$

At baseline, the rate of prescribing errors was 2.1%. This corresponds to a DPMO of 20,532.0197, indicating a relatively high frequency of errors within the prescribing process. The Sigma level, calculated at 3.5, reflects the process's capability, with higher values indicating fewer defects. In reference to global patient safety activities, the dedication of health leaders, stakeholders, and organizations in healthcare and education was highlighted [20]. They established a target of minimizing severe, unnecessary medication-related harm by 50% over the next five years globally [20]. Accordingly, we set our aim to achieve a reduction in prescribing errors by 10%, setting a target error rate of ≤1.9%.

Analyze phase

**Figure 2:**
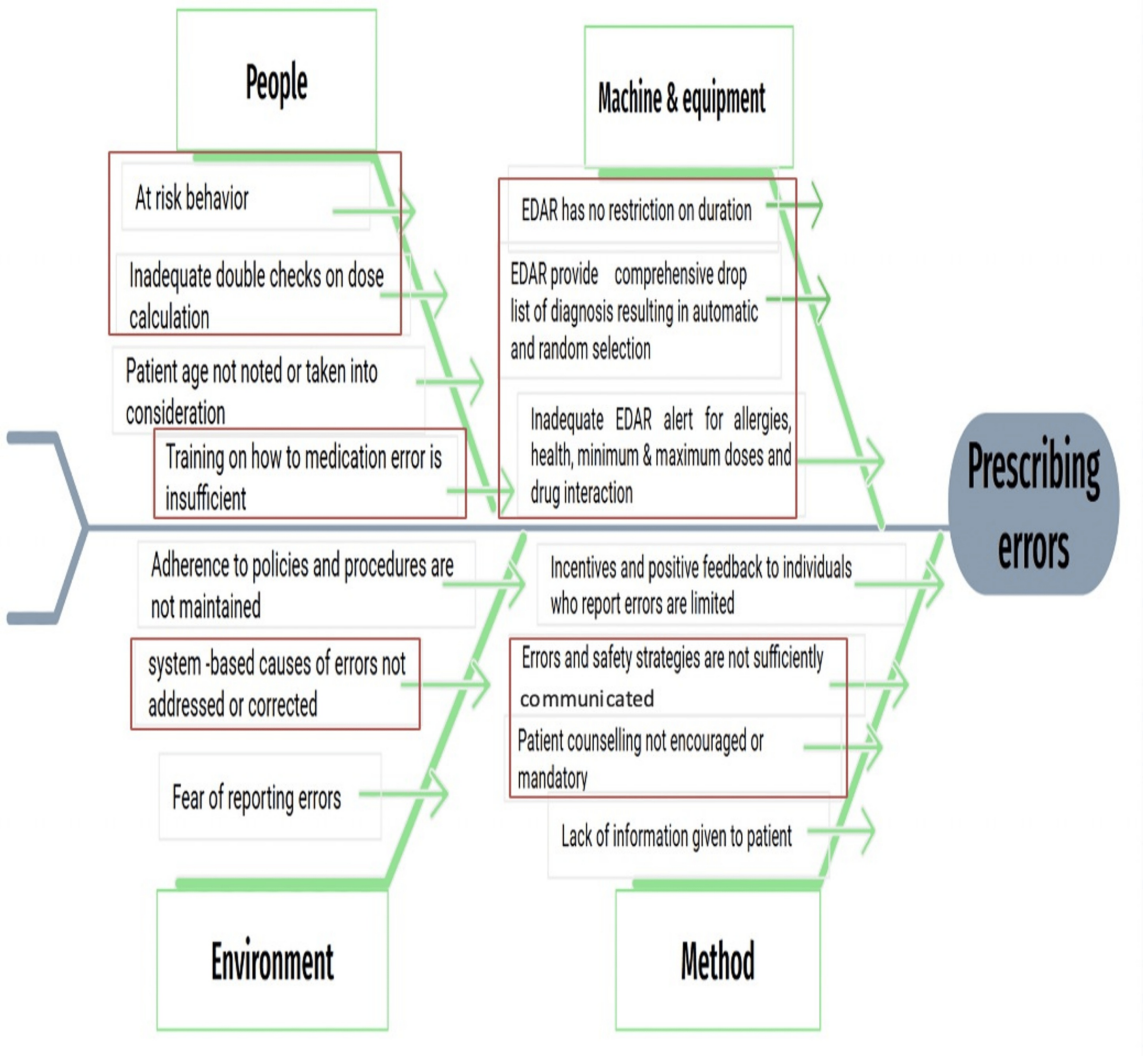
Fishbone analysis

In the Analyze phase, the Fishbone analysis was conducted as a structured way to identify the reasons for the problem (Figure 2). A major assessment of the current medication use process was initiated using the Institute for Safe Medication Practices (ISMP) Key Elements of the Medication Use System. This assessment referenced the guidebook titled "Assessing Risk and Opportunities for Change" to understand the underlying causes of the problem (Improving Medication Safety in Community Pharmacy) [21]. The assessment tool outlines the key elements (causes) related to medication use, facilitating the self-analysis of pharmaceutical errors and identifying necessary safety modifications (changes) to be implemented.

Improvement phase

The project started the improvement phase for six months from the end of March to September 2023. This was followed by the control phase for more than six months, which ended in March 2024. Improvement solutions have been set according to the previously identified causes in the fishbone (Figure 2). Therefore, the team sorted out a list of ideas or solutions. We employed the ISMP’s Reduction Strategies for Selecting the Change package. The ISMP’s Error Reduction Strategies employs a mix of higher and lower leverage strategies (ISMP’s Rank Order of Error Reduction Strategies) that focus on system issues and address human factors for those who work within that system (Table 1). Team members have discussed all these strategies and ranked the proposed solutions according to our issues.

**Table 1:** Rank order of error reduction strategies

Category/solution type	Leverage	Rank (implied)
Fail-safes and constraints	High Leverage	2
Forcing functions	High Leverage	2
Automation and computerization	High Leverage	2
Standardization and protocols	High Leverage	2
Redundancies	High Leverage	2
Reminders and checklists	High Leverage	2
Rules and policies	High Leverage	2
Education and information	Low Leverage	1

**Figure 3:**
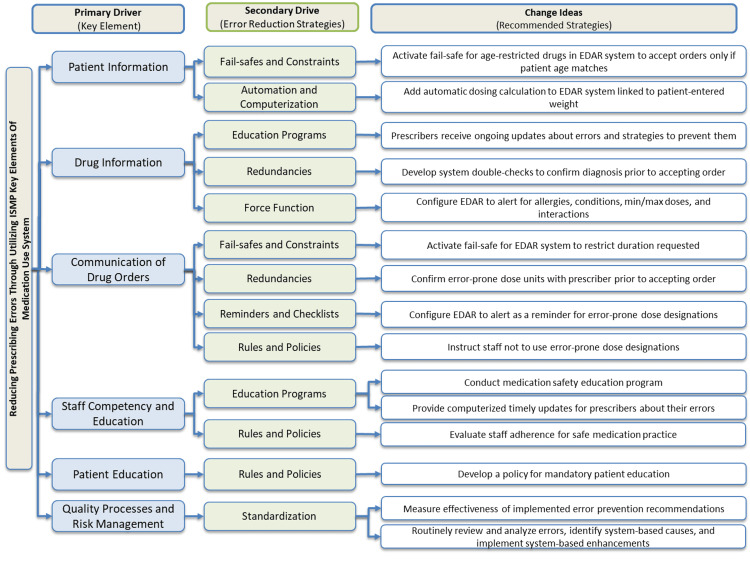
Driver diagram using ISMP ISMP: international safety medication practices; EDAR: Electronic Drug Administration Record

The outcome of this phase was the prioritization of the solutions by implementing a solution prioritization matrix. The team members voted by ranking the solution from 5-1 according to the time, impact, cost, and effort to be executed in the next phase (Table 2). The driver diagram (Figure 3) has been designed to summarize and integrate the aim, primary drivers (key elements), the secondary driver (error reduction strategy), and the ideas of the changes (recommended strategy).

**Table 2:** Solution prioritization matrix

Solutions	Time	Impact	Cost	Effort	Total
Forcing functions	5	5	5	5	20
Standardization and protocols	5	5	5	5	20
Fail-safes and constraints	4	5	5	4	18
Reminders and checklists	4	5	5	4	18
Automation and computerization	4	5	5	4	18
Rules and policies	4	5	5	4	18
Education and information	3	4	5	3	15
Redundancies	5	5	5	4	14
Purchasing an advanced pharmacy system	2	5	1	3	11

Control phase

A sustainability plan has been developed and implemented after a six-month improvement period to maintain the success of the improvement effort. Several key aspects were considered in the sustainability plan, including measurements, ownership, communication and training, hardwiring the change, and workload assessment. To ensure that the hard-earned gains are preserved, the plan has been communicated to all stakeholders, as outlined in Table 3 [22]. Through the careful implementation of best practices and ongoing monitoring of medication errors, a reduction from 2.1% to 1.3% was achieved, exceeding the target of ≤1.9%. Applying Six Sigma methodology and essential quality tools facilitated effective change management and progress tracking. To ensure ongoing sustainability and derive valuable insights from the data, a Power BI dashboard has been developed. This tool supports easy data visualization, real-time monitoring, customizable reporting, interactive analysis, transparent communication, and performance tracking. By leveraging Power BI (Microsoft Power BI), the objective is to sustain project outcomes and enhance patient safety within medication management processes.

**Table 3:** Sustainability planning worksheet: Institute for Healthcare Improvement, 2023 KPI: key performance indicator; CQI: continuous quality improvement; PS: patient safety; EDAR: Electronic Drug Administration Record; IT: Information Technology; QI: quality improvement

Area for consideration	What	Who	When
Measurements	We will continue measuring the inpatient prescribing errors KPI and the types of errors on the dashboard. Regular assessment of the risks and opportunities to change	Medication Safety Committee, and CQI and PS Medication Safety Committee	October 2023; annually
Assessment of the workload	Decrease the workload by automatic reporting. Extracting the medication errors from the EDAR system	IT	December 2023
Communication and training	Showing and sharing the success story for the QI through storyboard dissemination. Develop a policy for mandatory education and ensure dissemination of information (online, onsite)	Medication Safety Committee (Safety Officer) and medical admin	November 2023 and July 2023
Hardwiring the change	Automatic calculation in the EDAR system to facilitate error identification. Evaluate staff knowledge and adherence to safe medication practice	IT and medical admin	May 2023 to August 2023
Ownership	Continue in the structure of medication errors analysis and report from the medication safety committee to be reviewed by the medical admin monthly	Medication Safety Committee	Monthly

## Results

**Figure 4:**
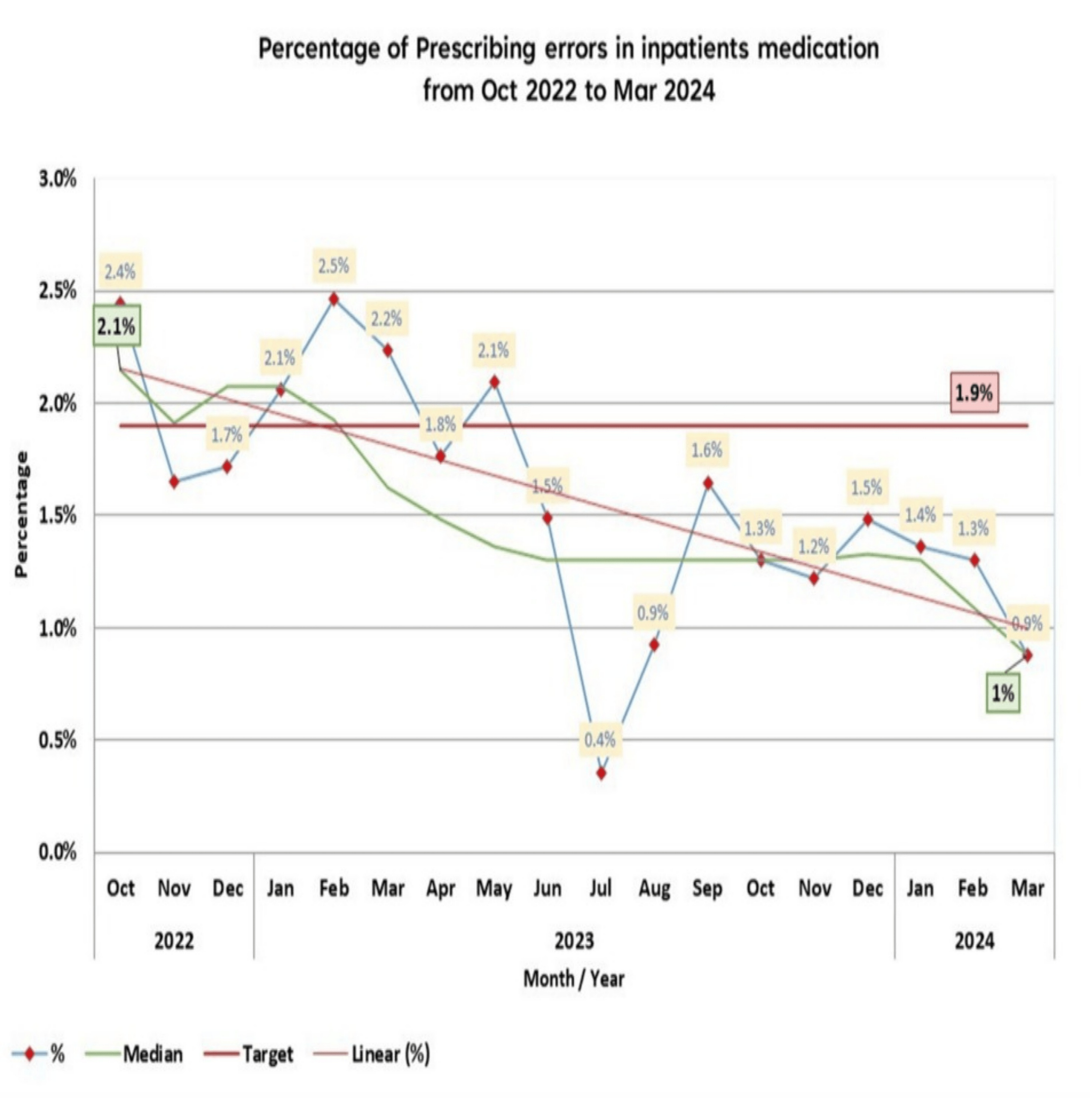
The outcome measures

The aim is to reduce the percentage of inpatient prescribing errors in the EDAR pharmacy system from a baseline of 2.1% to ≤1.9% within six months. The primary outcome measure is the percentage of prescribing errors for the inpatient pharmacy, which indicates a significant reduction of 0.8% across all inpatient prescriptions. During the project, spanning from April 2023 to March 2024, the percentage of prescribing errors decreased below the target of ≤1.9%, achieving a rate of 1.3% within 12 months of implementation. The accompanying line chart illustrates a consistent reduction in the median calculation, which declined from 2% to 1% (Figure 4).

Moreover, the improvement is reflected in the sigma level calculation, which increased from 3.5 to 3.7, corresponding to a DPMO of 13,760.29, close to the 13,900 mark. The overall mean score showed a slight improvement of approximately 0.2, with the average result rising from 1.2 before the application of the QI project to 1.4 following the improvements (Table 4). However, it is essential to recognize that balancing metrics may not always accurately represent project activity. Indirect relationships may exist, and various factors outside the specific interventions of the QI project can influence patient experience scores. External factors, such as organizational changes or seasonal variations, may impact the outcomes. Additionally, the relationship between the actions of the QI project and patient experience results is typically complex, with interventions not necessarily leading to immediate increases in satisfaction levels.

**Table 4:** Average of patient experience before and after the intervention calculated from the total overall score of inpatient services

Year	Month	Score	Average
2022	October (Before)	93.06	1.2833
	November (Before)	89.42	
	December (Before)	91.64	
2023	January (Before)	90.01	
	February (Before)	89.45	
	March (Before)	88.97	
	April (After)	91.81	1.432361
	May (After)	87.29	
	June (After)	89.19	
	July (After)	89.41	
	August (After)	87.45	
	September (After)	90.52	
	October (After)	93.06	
	November (After)	92.21	
	December (After)	90.59	
2024	January (After)	88.4	
	February (After)	89.2	
	March (After)	89.9	

The process measures the percentage of physicians who successfully completed medication safety training and education assessments, aiming to evaluate the effectiveness of the education program. Data collection for this measure commenced in June 2023, as illustrated in Table 5, demonstrating consistent performance across the various process measures presented in the accompanying graphs.

**Table 5:** Total points of distribution (training)

Number of respondents	Points scored	Average	Median	Range
1	50	81.49/100	85/100	50-100
2	55			
2	60			
2	65			
4	70			
10	75			
13	80			
14	85			
16	90			
6	95			
3	100			

**Figure 5:**
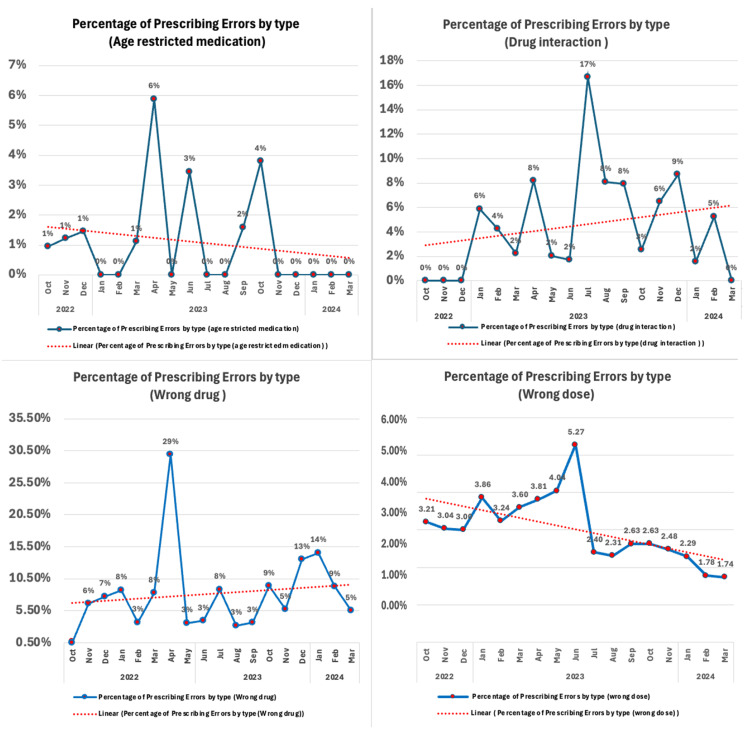
Process measure of prescribing errors by different types

All process measures concerning medication errors, categorized by type, including wrong drug, age-restricted medication, wrong dose, and drug interaction errors, were monitored due to their critical impact on patient safety (Figure 5). Run charts were employed to identify trends in medication errors over the duration of the QI initiative. Analysis of the graphs indicates a downward trend in errors related to age-restricted medications and wrong doses. Conversely, the trends for wrong drug and drug interaction errors exhibited slight increases among all inpatient prescriptions. Additionally, an analysis was performed to evaluate the effectiveness of physician training in safety program courses. The physicians who passed the training assessment exam achieved a median score of 85 and an average score of 81.4 (out of 100 points).

**Figure 6:**
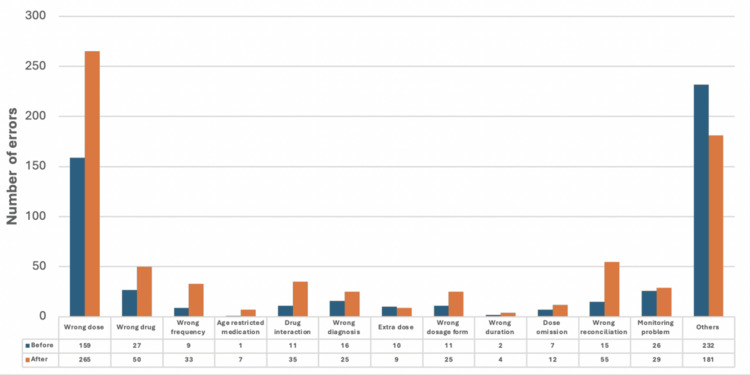
Prescribing errors by types before and after intervention

An additional analysis was conducted to assess and monitor various types of inpatient prescribing errors by number. The results displayed significant improvement across several error categories, as depicted in Figure 6. Wrong diagnosis, extra dose, wrong dosage administration route, incorrect duration, monitoring issues, and other errors exhibited substantial reductions in prescribing errors following the implementation of the project intervention.

## Discussion

The findings of this study now highlight the need for interventions to mitigate errors, like the approach taken by other studies. For example, Almalki et al. emphasize that prescription errors account for 44.4% of medication errors in Saudi Arabian hospitals, underscoring the urgency for robust strategies like those employed in this project [11]. Moreover, the systematic review conducted by Tobaiqy and MacLure supports the notion that improper dosing and prescribing errors are prevalent, advocating for comprehensive interventions to address these issues [23].

Our study interventions showed a significant reduction in prescribing errors, with a decrease of 0.8% across all inpatient prescriptions. In comparison, a study conducted in India by George et al. reported a larger reduction of 11.7% [18]. The project's primary focus was on prescribing errors caused by age-restricted medication, wrong dose, wrong drug, and drug interactions, which were the most affected by the major system change implemented during our project interventions, as shown in the process measures. In Figure 5, the errors related to the wrong drug did not achieve a high reduction. Conversely, age-restricted errors demonstrated a significant reduction, reaching 0% from the previous highest point, which was 6% before the QI intervention. Despite the clear downward trend in wrong dose prescribing errors, the outcome of the drug interaction errors showed fluctuating decreasing consecutive data points.

Furthermore, it has demonstrated a reduction in prescribing errors and ongoing challenges, such as the need for more flexible educational programs and the integration of advanced technologies, which highlight areas for future improvement. The necessity for adaptive strategies is also supported by research indicating that medication errors often result from systemic issues, such as inadequate communication and training gaps [5]. While the study made significant progress in reducing prescribing errors and enhancing the EDAR system, these findings underscore the significance of continued evaluation and adaptive generation of strategies to sustain and build on the initial progress in medication safety. This study successfully transformed the in-house developed EDAR system from an inhibitor into an enabler. Initially limited in capability, the system has been modified to provide a user-friendly interface for prescribers. However, significant challenges remain, particularly in integrating the system with medical laboratory data.

The flexibility and dedication demonstrated by pharmacy and IT staff played a crucial role in the successful implementation of the project, despite deviations from the original timeline. The ability to adapt and refine the plan as needed has proven to be a vital skill that enhanced the overall success of the initiative.

A culture of safety emerged as a significant enabler throughout the project. Patient safety was treated as a core value rather than a transient priority, acknowledging that priorities can shift over time. This collective commitment to safety fostered a dedication to error prevention within the quality project. Despite facing resource constraints, the willingness, accountability, and commitment of team members and hospital staff were pivotal in achieving the project’s goals. This QI initiative demonstrates that investing in an enhanced pharmacy system is a reliable indicator of success. By optimizing pharmacy systems, healthcare institutions can improve patient care outcomes, enhance medication accuracy, decrease errors, and streamline operations. Feedback from stakeholders, including patients, doctors, nurses, and pharmacists, provided valuable insights into the project’s success. Many users reported that the system upgrades have been well-received and positively impacted daily operations and patient care. Notably, these changes improved communication, made the system more user-friendly, and, in some instances, facilitated more informed decision-making for better patient outcomes.

Several limitations can be analyzed in this study, which may affect the overall findings and generalizability of the results. First, the reliance on self-reported data from stakeholders introduces potential biases, as individuals may have different perceptions of the system's effectiveness and usability. However, the inclusion of pharmacists in the data collection process strengthened the reliability of error reporting. Pharmacists are well-positioned to identify medication-related issues and are often involved in reviewing prescribing practices. As highlighted by Phansalkar et al., pharmacists demonstrated the highest level of thoroughness when conducting chart reviews, making their role critical in ensuring the accuracy and completeness of medication error detection [24]. Additionally, the limited duration of the study may not capture the long-term impacts of the implemented changes on medication errors and patient safety outcomes.

The study could have benefited from being a longitudinal study to compare better and assess the long-term impact of the interventions. Resource constraints, particularly in terms of staffing and IT support, also posed challenges to the timely execution of the project, potentially impacting the thoroughness of training and system integration. Furthermore, while the project emphasized a culture of safety, the extent to which this cultural shift has been sustained beyond the project’s implementation phase remains uncertain. Finally, the complexity of integrating the EDAR system with existing medical laboratory data continues to present challenges that were not fully addressed during the project, potentially limiting the comprehensiveness of the medication management process. This study adds to the expanding body of evidence that supports system-based approaches for reducing prescribing errors.

The structured approach of Six Sigma used in our project can serve as a model for similar QI projects. Clinically, the study resulted in significant reductions in important prescribing errors, underlining the need to optimize electronic prescribing systems and encouraging collaboration among physicians, pharmacists, and nurses to guarantee medication safety. Future improvements should expand to other phases of the medication-use process, and utilizing machine learning and data mining could help identify and address prescribing errors proactively. Also, we can consider deep learning techniques for data complex patterns. Therefore, we could apply interventions in the system based on prediction, such as providing decision support to prescribers, alerting for high-risk medications, or suggesting alternative medications.

## Conclusions

In conclusion, this study highlights the effectiveness of the Six Sigma methodology in addressing medication errors within the EDAR system at the Armed Forces Hospital in Dhahran, Saudi Arabia. Implementing targeted interventions led to a significant reduction in prescribing errors, improving patient safety and the overall quality of care. By transforming the previously limiting EDAR system into a user-friendly enabler, the project not only streamlined medication management processes but also fostered a culture of safety that prioritizes patient well-being above all else. While the project faced several challenges, including resource constraints and integration issues with medical laboratory systems, the commitment and flexibility of the pharmacy and IT teams proved essential in overcoming these obstacles. The positive feedback from stakeholders, patients, physicians, nurses, and pharmacists, underscored the success of the system upgrades and their impact on daily operations and patient care.

Future research should focus on long-term evaluations of the implemented changes to assess their sustainability and ongoing effectiveness. Additionally, exploring advanced technological solutions, such as machine learning and data mining, may further enhance the identification and prevention of medication errors. By continually investing in and optimizing pharmacy systems, healthcare institutions can make significant strides in improving medication accuracy, reducing errors, and ultimately ensuring safer patient outcomes. This study serves as a model for other healthcare organizations aiming to enhance their medication management processes and underscores the critical importance of a culture of safety in delivering high-quality care.

## Appendices

Appendix 1

**Table 6:** Percentage of medication errors by stage involved in quarter 4 of 2022

Medication process stage	Percentage
Prescribing	83%
Transcribing	3%
Preparing	5%
Dispensing	6%
Administration	3%
Monitoring	1%

Appendix 2

**Table 7:** Percentage of errors in prescribing for inpatient and outpatient quarter 4 (2022) to quarter 1 (2023)

Setting	Percentage
Inpatient	2.10%
Outpatient	0.66%

Appendix 3

**Table 8:** Inpatient and outpatient prescribing errors from October 2022 to March 2023

Patient types	Number of prescriptions	Number of prescribing errors	% Distribution of prescribing errors	% Prescribing errors
Inpatient	25,375	521	0.40%	2.1%
Outpatient	104,480	693	0.53%	0.66%
Total	129,855	1,214	0.93%	-
